# Isolation and Identification of a Novel Anti-protein Aggregation Activity of Lignin-Carbohydrate Complex From *Chionanthus retusus* Leaves

**DOI:** 10.3389/fbioe.2020.573991

**Published:** 2020-09-25

**Authors:** Wenhui Pei, Zhefan Stephen Chen, Ho Yin Edwin Chan, Liming Zheng, Chen Liang, Caoxing Huang

**Affiliations:** ^1^Guangxi Key Laboratory of Clean Pulp & Papermaking and Pollution Control, Guangxi University, Nanning, China; ^2^Co-Innovation Center of Efficient Processing and Utilization of Forest Resources, Department of Bioengineering, Nanjing Forestry University, Nanjing, China; ^3^Nexus of Rare Neurodegenerative Diseases, School of Life Sciences, Faculty of Science, The Chinese University of Hong Kong, Shatin, China; ^4^Gerald Choa Neuroscience Centre, The Chinese University of Hong Kong, Shatin, China; ^5^State Key Laboratory of Pharmaceutical Biotechnology, Department of Sports Medicine and Adult Reconstructive Surgery, Nanjing Drum Tower Hospital, The Affiliated Hospital of Nanjing University Medical School, Nanjing, China

**Keywords:** *Chionanthus retusus* leaves, lignin-carbohydrate complex, antioxidant, anti-protein aggregation, reactive oxygen species

## Abstract

Lignin-carbohydrate complex (LCC) is the biological macromolecule that has been demonstrated to exert multiple biological functions, including antioxidant, anti-inflammation and anti-tumorigenesis, which support its broad application in the bioengineering field. However, it remains elusive the involvements of LCC in human neurological disorders, especially those with the overproduction of reactive oxygen species (ROS), such as spinocerebellar ataxias (SCAs). In this study, we found a previously undetermined anti-protein aggregation activity of LCC. Initially, two individual LCC preparations and carbohydrate-free lignin were isolated from the water-extracted waste residues of *Chionanthus retusus* (*C. retusus*) tender leaves. The chemical compositional analysis revealed that lignin (61.5%) is the predominant constituent in the lignin-rich LCC (LCC-L-CR), whereas the carbohydrate-rich LCC (LCC-C-CR) is mainly composed of carbohydrate (60.9%) with the xylan as the major constituent (42.1%). The NMR structural characterization showed that LCC-L-CR preparation is enriched in benzyl ether linkage, while phenyl glycoside is the predominant type of linkage in LCC-C-CR. Both LCC and lignin preparations showed antioxidant activities as exemplified by their abilities to scavenge free radicals in cultured mammalian cells and ROS in zebrafish. We further demonstrated a pronounced capability of LCC-L-CR in inhibiting the aggregation of expanded Ataxin-3, disease protein of SCA type 3, in human neuronal cells. Taken together, our study highlights the antioxidant and novel anti-protein aggregation activities of the *C. retusus* tender leaves-derived LCC.

## Introduction

*Chionanthus retusus* Lindl. et Paxton (*C. retusus*) contains various types of deciduous trees that are distributed across tropical and subtropical Asian countries, including China, Japan, North Korea, and South Korea (Arias et al., 2011). The tender leaf from *C. retusus* has been widely used in preparing medicinal tea or consumed beverages due to its abundance in polyphenols, flavonoids, and functional polysaccharides (Lee et al., 2019). Generally, the hot water (90–100°C) extraction (or “steeping”) predominantly releases water soluble constituents (tea polyphenols, theophylline, amino acids, and vitamins) from roasted tender leaves, leaving the majority of polysaccharides (cellulose and hemicellulose) and lignin in the residual solids (Liu et al., 2018; Fu et al., 2020). These residual solids are initially considered as agricultural residues for disposal or burning, which can lead to severe environmental contamination and air pollution. However, recently, emerging evidence has demonstrated that both polysaccharides and lignin from residual solids are invaluable resources for preparing multiple kinds of bio-materials and bio-chemicals using a biorefinery-type process (Chen D. et al., 2018; Zhao et al., 2018; Wu et al., 2019; Pei et al., 2020; Wei et al., 2020). Therefore, an ideal and greener waste management alternative should be considered to reuse the residual solids from *C. retusus* leaves.

For *C. retusus*, cellulose, hemicellulose and lignin are physically and chemically interlinked within the cell wall (Ten and Vermerris, 2013; Song and Hong, 2020). Anatomically, lignin and carbohydrate (mainly xylan) that are covalently linked via benzyl ethers, benzyl esters and phenyl glycoside moieties are referred to as lignin-carbohydrate complexes (LCCs) (Dong et al., 2020; Lin et al., 2020; Wang et al., 2020). Compared to neat lignin, the carbohydrate portions in LCCs could facilitate their recognition by cells, thus improving the cellular compatibilities of LCCs (Jiang et al., 2010). The LCCs function as oxidative stress suppressors and immune stimulants (Huang et al., 2019; Jiang et al., 2019). For example, Zhang et al. (2018) used the hot-water extraction liquor from spruce wood to isolate the LCC fraction, which exhibits pronounced activity toward scavenging free radicals *in vitro*. Dong et al. (2020) demonstrated the isolation of carbohydrate-rich LCC preparations from different biomass, and they found that these LCCs possess strong capabilities toward scavenging intracellular and endogenous reactive oxygen species (ROS). Given its antioxidant activity, LCC has been widely implicated in executing anti-tumor, anti-microbial, anti-parasite, and anti-viral functions (Sakagami et al., 2005, 2010). Therefore, we are intended to investigate the potential biological functions of LCCs isolated from the water-extracted residual solids of *C. retusus* tender leaves.

The excessive production of ROS in cells leads to oxidative stress, which contributes to a group of human disorders including cancer, diabetes, inflammatory diseases, and neurodegenerative diseases (Waris and Ahsan, 2006). The abnormal genetic expansion of polyglutamine (polyQ) tract is causative for a specific group of neurodegenerative disorders known as polyQ diseases, which include Huntington’s disease and several subtypes of spinocerebellar ataxias (SCAs) (Lieberman et al., 2019). As the pathological hallmark of polyQ diseases, the formation of polyQ protein aggregates in neurons impairs a range of normal biological functions and contributes to polyQ neurodegeneration (Adegbuyiro et al., 2017). A series of chemical inhibitors, including small molecule compound (Hong et al., 2019), synthetic peptide (Nagai et al., 2003) and natural product extract (Walter et al., 2014), have been reported to ameliorate polyQ cytotoxicity via lowering the level of polyQ protein aggregates. Moreover, several lines of evidence have demonstrated the neuroprotective roles of antioxidants in polyQ diseases (Wu et al., 2017; Essa et al., 2019). However, no reports have ever demonstrated the potential relationship between LCC and polyQ diseases. It would therefore be interesting to investigate whether the LCCs isolated from *C. retusus* leaves would elicit any modulatory effect on polyQ pathogenesis.

In the current study, we used hot-water extracted residual solids from *C. retusus* tender leaves as feedstock to isolate two independent carbohydrate (LCC-C-CR) and lignin enriched (LCC-L-CR) LCC preparations, the carbohydrate-free lignin preparation (Lignin-CR) was isolated simultaneously. Their chemical structures were further comprehensively characterized using size exclusion chromatography and NMR. The *in vitro* mammalian cell and *in vivo* zebrafish models were used to evaluate the antioxidant activities of LCC-C-CR, LCC-L-CR, and Lignin-CR, and all of them showed scavenging capabilities toward free radicals and endogenous ROS. Moreover, we found that treatment of LCC-L-CR suppresses polyQ protein aggregation in neuronal cells. Taken together, our study is the first report to demonstrate the novel anti-protein aggregation activity of LCC. This contributes to the broaden application of LCC in the biological and bioengineering fields, and further suggests the potential benefits of LCC on protecting neuronal dysfunctions in human protein misfolding diseases.

## Materials and Methods

### *C. retusus* Tender Leaves

Tender leaves from *C. retusus* tree (5 years old, locked in Suqian, Jiangsu, China) were collected in the spring of 2019. The fresh leaves were dried at 90°C for 5 h before immediately ground into the powder with particle sizes between 20 and 40 mesh. The obtained powder was stored at 4°C for further study and analysis.

### Isolation and Purification of LCC and Lignin Preparations From *C. retusus* Leaf Powder

The *C. retusus* tender leaf powder was steeped in hot water (95°C) for 2 h in order to imitate the process of tea beverage preparation. After steeping, the hot-water extracted residual solid (ERDS) were separated and dried at 40°C, followed by the extraction with benzene/ethanol (2:1, v/v) in a Soxhlet extractor for 12 h to remove pigments and alcohol-soluble substances.

Lignin-rich LCC, carbohydrate-rich LCC and lignin fractions were then prepared from the extracted ERDS according to the classical method described by Bjoörkman (1957). Briefly, ERDS (∼4 g) was subjected to planetary ball milling in a 100 mL ZrO_2_ bowl with 25 ZrO_2_ balls at 600 rpm for 5 h. Milled ERDS was then extracted using aqueous dioxane (96%, v/v) at solid to liquid ratio of 1:10 (g/mL) at room temperature for 24 h. This process was repeated three times, and then the extracted solutions were combined and evaporated under vacuum at 40°C. The lignin-rich LCC and lignin in the extracted solutions were also purified according to the standard protocol in Bjoörkman’s (1957) work. Next, the residual matter after triple dioxane extraction was washed with distilled water to remove all traces of dioxane. From this washed matter, the carbohydrate-rich LCC preparation was isolated using an acetic acid solution (50%, v/v) and purified according to the standard protocol in Bjoörkman (1957). The lignin-rich LCC, carbohydrate-rich LCC and lignin fractions from ERDS of *C. retusus* were termed LCC-L-CR, LCC-C-CR and Lignin-CR, respectively.

### Chemical Compositional Analysis of *C. retusus*, LCC and Lignin Preparations

The chemical compositions (carbohydrates and lignin) of raw *C. retusus* tender leaf, hot water extracted *C. retusus* tender leaf, LCC and lignin preparations were analyzed according to the procedures developed by the National Renewable Energy Laboratory (Sluiter et al., 2011). The monosaccharides in all samples were analyzed by high-performance anion exchange chromatography system (Dionex ISC 5000, Sunnyvale, CA, United States), which was equipped with the PA10 (2 × 250 mm) column and pulsed amperometric detector (Zhou and Xu, 2019). The amount of polyphenols in the LCC and lignin preparations was determined according to Folin–Ciocalteu method using gallic acid as a calibration standard by UV-vis spectrophotometer at 745 nm (Singleton and Rossi, 1965). The samples were prepared according to our previous work (He et al., 2020).

### Molecular Weights Analysis of LCC and Lignin Preparations

Weight-average (M_w_) and number-average (M_n_) molecular weights of LCC and lignin preparations were measured using a gel permeation chromatography (GPC) system equipped with the PL-gel 10 mm and mixed-B 7.5 mm i.d. column and an ultraviolet detector. The samples were fully dissolved in tetrahydrofuran (THF) at the concentration of 1 mg/mL. Monodisperse polystyrenes (Sigma) with 1320, 9200, 66,000, 435,500 g/mol were used as the standards to calculate the molecular weights. The analysis procedures were carried out at 35°C with THF as eluent with flowing rate of 1 mL/min.

### NMR Analysis of LCC and Lignin Preparations

Complete structural information (sub-units, lignin linkages, and lignin-carbohydrate linkages) of both LCC and lignin preparations was determined using a Bruker AVANCE 600 MHz spectrometer, in which the quantitative ^13^C NMR and 2D-HSQC NMR spectra were obtained using dimethyl sulfoxide-d_6_ (DMSO-d_6_) as the solvent. The sample preparation steps for NMR analysis were described previously (Dong et al., 2020). For ^13^C NMR analysis, the acquisition parameters were 25°C, 90° pulse width, 1.7 s relaxation, and 1.2 s of acquisition time. For 2D-HSQC NMR analysis, 160 transients (scans per block) were acquired using 1024 data points in F2 (^1^H) dimension with an acquisition time of 53 ms and 256 data points and F1 (^13^C) dimension with an acquisition time of 5.14 ms. The amounts (per 100 Ar (C_900_)) of lignin linkages and lignin-carbohydrate linkages in both LCC samples were calculated from a combination of quantitative ^13^C and 2D HSQC NMR according to Zhang and Gellerstedt (2007) using the following formulas:

$$\begin{array}{c} \text{Ix}={\text{2DI}}_{\text{x}}/{\text{2DI}}_{78-90/2.5-6.0}\times \\ {}^{13}{\text{C}}_{\text{78-90}}{/}^{13}{\text{C}}_{103-163}\times 600\\ \text{BE}=2{\text{D}}_{\text{BE}}/2{\text{D}}_{78-90/3.0-5.7}\times \\ 13{\text{C}}_{96-103}{/}^{13}{\text{C}}_{103-163}\times 600\\ \text{PhGlc}=2{\text{D}}_{\text{PhGlc}}/2{\text{D}}_{96-103/3.8-5.5}\times \\ {}^{13}{\text{C}}_{96-103}{/}^{13}{\text{C}}_{103-163}\times 600\\ \gamma -\text{Est}=2{\text{D}}_{\text{Est}}/2{\text{D}}_{58-65/2.5-5.0}\times \\ {}^{13}{\text{C}}_{58-65}{/}^{13}{\text{C}}_{103-163}\times 600 \end{array}$$

In the formulas, I_x_ is the integral value of *a* position signal in lignin linkages (β-O-4, β-β, and β-5) in the 2D spectra region. BE, PhGlc, and γ-Est are the amounts of benzyl ether, phenyl glycoside, and γ-ester LCC linkages. During the integration process, all the integration was conducted at the same contour level.

The contents of aliphatic hydroxyl and phenolic hydroxyl in LCC and lignin preparations were determined by quantitative ^31^P NMR using a Bruker AVANCE 600 MHz spectrometer, in which endo-*N*-hydroxy-5-norbornene-2, 3-dicarboximide (e-NHI) was used as the internal standard. The samples [40 mg of solid was dissolved in 0.5 mL of anhydrous pyridine/CDCl_3_ mixture (1.6:1, v/v)] were prepared according to our previous report (Huang et al., 2017). The acquisition parameters with a total number of 256 scans were used to obtain the spectra in ^31^P NMR analysis.

### Preparation of LCC and Lignin Stock Solutions for Biological Experiments

The LCC-C-CR, LCC-L-CR and Lignin-CR samples were dissolved in dimethylsulfoxide (DMSO) to prepare the stock solutions at 25 mg/mL. The solutions were briefly sonicated (30 s) in ultrasonic bath (DC-80H, Healthcare Technologies) to dissolve all the matters before being used in the subsequent biological experiments.

### Antioxidant Activity Analysis of LCC and Lignin Preparations

Both *in vitro* mammalian cell and *in vivo* zebrafish experimental models were used to evaluate antioxidant activities of LCC and lignin preparations. The measurements of scavenging capabilities on 2,2-diphenyl-1-picryl-hydrazyl (DPPH) and superoxide anion (O_2_^•–^) radicals in RAW 264.7 cells, and endogenous ROS in zebrafish were carried out as described previously (Dong et al., 2020). For DPPH scavenging assay, the RAW 264.7 cells were treated with LCC or lignin preparations at 0.005 mg/mL, 0.01 mg/mL, 0.02 mg/mL, 0.05 mg/mL, 0.1 mg/mL, and 0.2 mg/mL. For O_2_^•–^ scavenging assay, the RAW 264.7 cells were treated with LCC or lignin preparations at 0.1 mg/mL, 0.2 mg/mL, 0.4 mg/mL, 0.6 mg/mL, 0.8 mg/mL, 1.0 mg/mL, and 2.0 mg/mL. For ROS scavenging assay, the zebrafish was treated with LCC or lignin preparations at 200 μg/mL. All animal experiments were performed following the guiding principles for the care and use of animals approved by the animal ethics committee of Nanjing Drum Tower Hospital, The Affiliated Hospital of Nanjing University Medical School (Permit number: 2019AE01018).

### Cytotoxicity Evaluation of LCC and Lignin Preparations

The cytotoxicities of LCC-C-CR, LCC-L-CR, and Lignin-CR were measured using a CytoTox-Glo^TM^ Cytotoxicity Assay kit (G9290, Promega) according to the manufacturer’s instructions. Briefly, the human neuroblastoma SK-N-MC cells were cultured in 96-well plates and treated with LCC or lignin preparations at 25 μg/mL, 50 μg/mL, 100 μg/mL, 200 μg/mL, 400 μg/mL, and 800 μg/mL. The treatment lasted for 24 h. The luminescent intensities were measured on a FLUOstar Omega Microplate Reader (BMG LABTECH). The percentage of live cells was calculated by the following equation:

$$\text{Percentage of live cells (}\%)=\frac{{\text{luminescence}}_{\text{total}}-{\text{luminescence}}_{\text{dead}}}{{\text{luminescence}}_{\text{total}}}\times \text{ 100\%}$$

### Generation of *pAcGFP-ATXN3Q71* Plasmid

To generate *pAcGFP-ATXN3Q71*, the *ATXN3Q71* DNA fragment amplified from *pEGFP-ATXN3Q78* was subcloned into the *pAcGFP-C1* vector (632470, Clontech) using *Kpn*I and *Bam*HI (Chen Z. S. et al., 2018; Chen et al., 2019). In contrast to EGFP, which is prone to form dimers, AcGFP is a monomeric green fluorescent protein with equivalent brightness (Zacharias et al., 2002; Gurskaya et al., 2003). When AcGFP-ATXN3Q71 was expressed in our cell model, the number of ATXN3Q71 protein aggregates in each cell could be clearly detected and easily counted, while this is more difficult to achieve in EGFP-ATXNQ78-expressing cells (Hong et al., 2019).

### SK-N-MC Cell Culture and Transfection

Human neuroblastoma cell line SK-N-MC (HTB-10^TM^, American Type Culture Collection) was cultured using Dulbecco’s Modified Eagle’s Medium (11995065, Thermo Fisher Scientific) supplemented with 10% Fetal Bovine Serum (F7524, Sigma-Aldrich) and 1% Antibiotic-Antimycotic solution (15240062, Thermo Fisher Scientific). The cells were maintained in a 37°C humidified cell culture incubator supplemented with 5% CO_2_. Lipofectamine 2000 (11668019, Thermo Fisher Scientific) was used in plasmid transfection. The 0.5 μg of *pAcGFP-ATXN3Q71* plasmid, together with 1 μL of Lipofectamine 2000 (11668019, Thermo Fisher Scientific) were used to transfect SK-N-MC cells, and the transfection lasted for 48 h.

### Fluorescence Imaging of ATXN3Q71 Protein Aggregates in SK-N-MC Cells

The SK-N-MC cells were cultured on coverslips (Marienfeld-Superior). Twenty-four hours post transfection, the cells were treated with LCC or lignin preparations at 100 μg/mL for another 24 h, prior to being harvested for the subsequent fluorescence imaging of ATXN3Q71 protein aggregates. Cells were fixed with 3.7% paraformaldehyde for 15 min followed by permeabilization with 0.1% Triton X-100 for another 15 min. The cell nuclei were stained with DAPI (62248, Thermo Fisher Scientific) at 25°C for 5 min. Fluorescence was acquired using a confocal microscope Zeiss LSM (Zeiss) and images were analyzed using Fiji software (Version 2.0.0-rc-69/1.52n, NIH).

### Statistical Analysis

The two-tailed, unpaired Student’s *t*-test, or Mann–Whitney *U* test was used for the comparison of two experimental groups. One-way ANOVA followed by *post hoc* Tukey’s test was applied to determine the difference between each experimental group when comparing three or more groups. ^∗^, ^∗∗^, and ^∗∗∗^ represent *p* < 0.05, *p* < 0.01, and *p* < 0.001, respectively, which are considered statistically significant. ns indicates no significant difference. GraphPad Prism (Version 8.1.2) was used for statistical analysis.

## Results and Discussion

### Compositional Analysis of *C. retusus* Tender Leaf and Its Derived Materials

We first aimed at investigating the detailed composition of polysaccharides (cellulose and hemicellulose) and lignin in *C. retusus* tender leaf and its derived materials. The raw *C. retusus* tender leaf (CR-TL), hot water extracted *C. retusus* tender leaf (WE-CR-TL), LCC and lignin preparations were used for the compositional analysis (Table 1). The results showed that polysaccharides were more abundant in CR-TL. Among the determined polysaccharides, cellulose (expressed as glucan) was the major component (42.1%), followed by hemicelluloses (26.1%), which was composed of xylan (21.1%), arabinan (2.5%), galactan (1.1%), and mannan (1.4%). The percentage of lignin in CR-TL was 19.5%, which is slightly lower than those in conventional hardwood (20–25%) and softwood (26–32%). We hypothesize that this reduction of lignin is likely due to the leaves being 1–3 months in age, which does not allow sufficient lignification to occur. We detected a similar composition of polysaccharides and lignin (according to National Renewable Energy Laboratory procedures) in WE-CR-TL, with the respective amounts of cellulose, hemicellulose and lignin were 49.5%, 18.4%, and 22.9%. The percentage of hemicellulose in WE-CR-TL (18.4%) was lower than what was detected in CR-TL (26.1%), indicating a labile portion of hemicellulose undergoes degradation after hot water extraction (Azhar et al., 2015; Gu et al., 2020).

**TABLE 1:** Chemical compositions of *C. retusus* tender leaf, hot water extracted *C. retusus* tender leaf, LCC and lignin preparations.

	Lignin (%)	Cellulose (%)	Hemicellulose (%)				Polyphenols (%)	Hydroxyl content (mmol/g)^b^	
			Xylan	Arabinan	Galactan	Mannan		Aliphatic	Phenolic
CR-TL^a^	19.5	42.1	21.1	2.5	1.1	1.4	/	/	/
WE-CR-TL^a^	22.9	49.5	17.4	0.8	0.1	0.1	/	/	/
LCC-L-CR^a^	61.5	6.7	23.1	0.9	0.3	0.8	2.1	3.95	1.35
LCC-C-CR^a^	31.9	9.9	42.1	6.3	1.5	1.1	1.7	4.12	1.01
Lignin-CR^a^	92.5	0.2	0.8	0.1	0	0	3.5	3.65	1.89

*^*a*^CR-TL, raw *C. retusus* tender leaf; WE-CR-TL, hot water extracted *C. retusus* tender leaf; LCC-L-CR, lignin-rich LCC from WE-CR-TL; LCC-C-CR, carbohydrate-rich LCC from WE-CR-TL; Lignin-CR, lignin from WE-CR-TL. ^*b*^Determined by quantitative ^31^P NMR.*

As shown in Table 1, we obtained two distinct LCC preparations, which were enriched in lignin (LCC-L-CR) and carbohydrate (LCC-C-CR), respectively. The carbohydrates in both LCC preparations comprise the same types of sugars, including glucan, xylan, arabinan, galactan, and mannan. Moreover, the types of sugars in our LCCs coincide with those in LCCs derived from hardwood and non-woody biomass (Dong et al., 2020). When looking into the amount of each type of sugars, we found that xylan is the predominant component in both LCC-L-CR (23.1%) and LCC-C-CR (42.1%). Meanwhile, the amounts of glucan (6.7% and 9.9%), arabinan (0.9% and 6.3%), galactan (0.3% and 1.5%), and mannan (0.8% and 1.1%) were relatively lower. The compositional analysis was also carried out on carbohydrate-free lignin preparation isolated from *C. retusus* tender leaf (Lignin-CR). As shown in Table 1, only trace amount of carbohydrate (1.1%) was detected, suggesting Lignin-CR as a relatively pure lignin sample.

Given the importance of polyphenols in contributing to the biological functions of biopolymers, the amount of polyphenols in LCC and lignin preparations were further measured (Table 1). We found a slightly higher amount of polyphenols in Lignin-CR (3.5%), when compared to LCC-L-CR (2.1%) and LCC-C-CR (1.7%). Next, quantitative ^31^P NMR was performed with the aim to unveiling the detailed contents of aliphatic and phenolic hydroxyl in LCC and lignin preparations. Both aliphatic and phenolic hydroxyl were detected in LCC-L-CR, LCC-C-CR and Lignin-CR, with the amount of aliphatic hydroxyl (ranging from 3.65 to 4.12 mmol/g) was higher than phenolic hydroxyl (ranging from 1.01 to 1.89 mmol/g).

### Molecular Weight Analysis of LCC and Lignin Preparations

Molecular weight estimations, including the weight-average molecular weights (M_*w*_), number-average molecular weights (M_n_) and polydispersity (M_w_/M_n_) of LCC and lignin preparations are summarized in Table 2. The M_w_ of LCC-C-CR (19870 g/mol) was higher than that of LCC-L-CR (9700 g/mol). A similar trend was observed for M_n_, with the values in LCC-C-CR and LCC-L-CR were 9800 g/mol and 6570 g/mol, respectively. Meanwhile, the Lignin-CR preparation showed lowest value of M_w_ (8820 g/mol) and M_n_ (4910 g/mol). The higher molecular weight identified in LCC-C-CR preparation may be due to the following two reasons: (1) the M_w_ of LCC-C-CR is indeed higher than that of LCC-L-CR; (2) the higher content of carbohydrate linked to lignin in LCC-C-CR alters the hydrodynamic volume of lignin, leading to the increase of apparent molar mass of lignin in GPC measurements (Sun et al., 2012). Moreover, LCC-C-CR, LCC-L-CR, and Lignin-CR exhibited narrow molecular weight distribution with relative polydispersity of 2.0, 1.5, and 1.8.

**TABLE 2:** The molecular weight (g/mol) and polydispersity of LCC-C-CR, LCC-L-CR and Lignin-CR.

	M_w_^b^	M_n_^b^	Polydispersity^c^
LCC-C-CR^a^	19870	9800	2.0
LCC-L-CR^a^	9700	6570	1.5
Lignin-CR^a^	8820	4910	1.8

*^*a*^LCC-C-CR, carbohydrate-rich LCC from WE-CR-TL; LCC-L-CR, lignin-rich LCC from WE-CR-TL; Lignin-CR, lignin from WE-CR-TL. ^*b*^M_*w*_, weight-average of molecular weight; M_*n*_, number-average of molecular weight. ^*c*^Polydispersity: M_*w*_/M_*n*_.*

### Structural Characterization of LCC and Lignin Preparations

To obtain more insights on the actual chemical structures in LCC and lignin preparations, the advanced spectroscopy of NMR with ^13^C NMR and 2D-HSQC were used. The quantitative ^13^C NMR spectra of both LCC preparations are shown in Supplementary Figure 1, and all signals in spectra were assigned according to previous studies (Huang et al., 2019; Dong et al., 2020; Shen et al., 2020). As demonstrated, the abundant signals in 153–103 ppm region were attributed to the syringyl units (S) and guaiacyl units (G) in LCC. In 90–50 ppm region, the signals were mainly from the substructures (β-O-4 and β-5) and carbohydrate. In addition, the signals that could be readily detected in 102–90 ppm were also attributed to the carbohydrate (Supplementary Figure 1). Unfortunately, the signals of lignin-carbohydrate linkages in LCC overlap with the carbohydrate signals from 102 to 90 ppm and thus cannot be resolved using this method (Balakshin et al., 2007).

**FIGURE 1:**
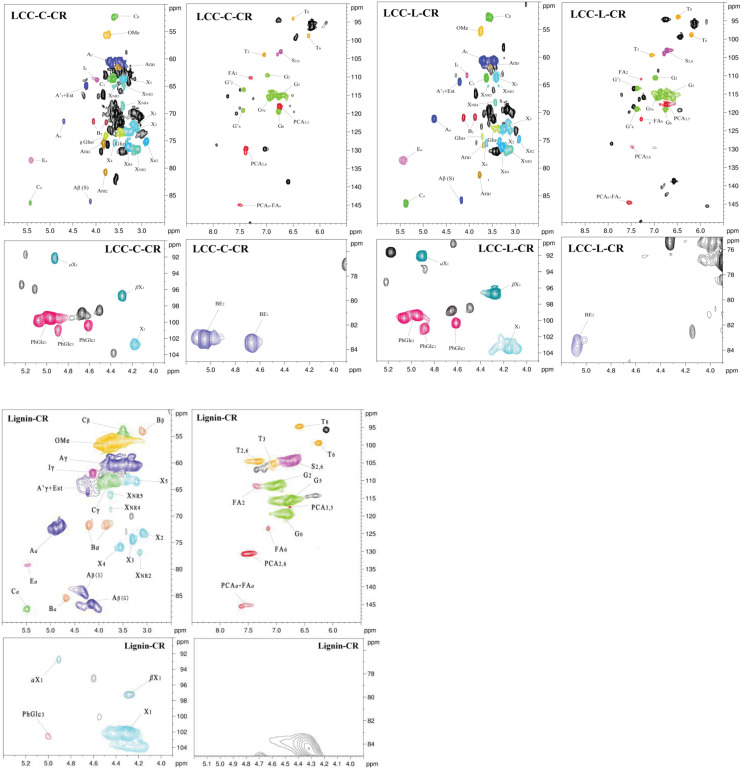
2D-HSQC NMR spectra of LCC-C-CR, LCC-L-CR, and Lignin-CR samples.

**FIGURE 2:**
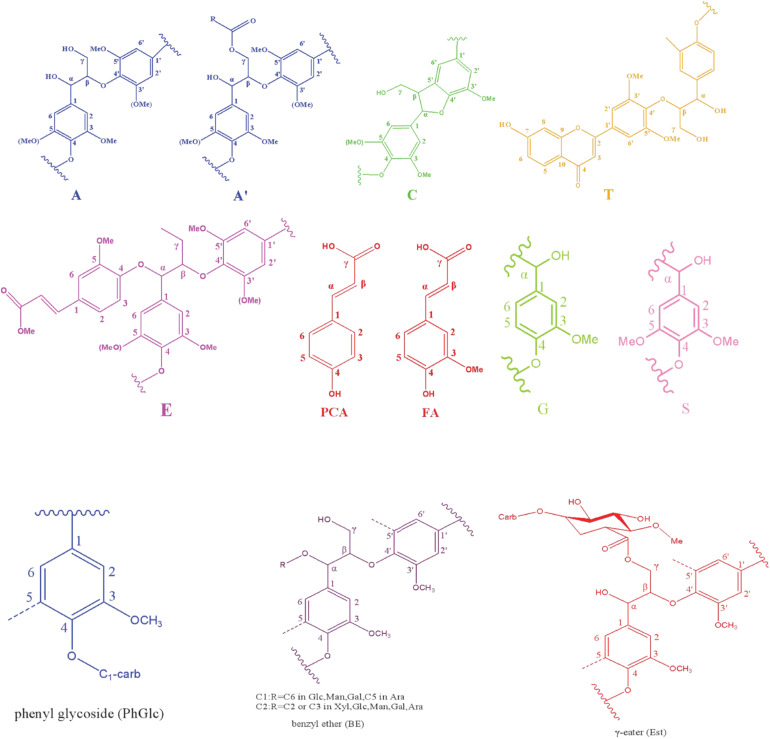
Main structures identified in the LCC preparations.

To overcome the issues of overlapping signals between lignin-carbohydrate linkages and carbohydrate in ^13^C NMR, the LCC and lignin preparations were further analyzed using 2D-HSQC NMR. The obtained spectra are shown in Figure 1. The main cross-signal assignments of the substructure and linkages in 2D-HSQC NMR spectra are summarized in Table 3, and their structures are graphically depicted in Figure 2. All signals from 2D-HSQC NMR spectra were assigned according to previous studies (Huang et al., 2019; Dong et al., 2020; Shen et al., 2020).

**TABLE 3:** Main cross-signal assignments of substructure and linkages in 2D-HSQC NMR spectra.

Labels	δ_C_/δ_H_	Assignment
**Lignin structure**		
C*_β_*	53.1/3.49	C*_β_*-H*_β_* in phenylcoumaran substructures (C)
-OCH_3_	55.9/3.73	C-H in methoxyls
A*_γ_*	59.6–60.8/3.37–3.72	C*_γ_*-H*_γ_* in β-O-4 substructures (A)
A′*_γ_*	63.6/4.36	C*_γ_*-H*_γ_* in γ-acylated β-O-4 substructures (A′)
E*_*a*_*	79.5/5.45	C*_*a*_*-H*_*a*_* in *a*,β-diaryl ether (E)
A*_β_*_(*S*)_	86.0/4.11	C*_β_*-H*_β_* in β-O-4 substructures linked to a S unit (A)
C*_*a*_*	86.8/5.49	C*_*a*_*-H*_*a*_* in phenylcoumaran substructures (C)
T_8_	94.2/6.59	C_8_-H_8_ in tricin (T)
T_6_	98.8/6.27	C_2,6_-H_2,6_ in tricin (T)
T_3_	106.1/7.07	C_3_-H_3_ in tricin (T)
S_2,6_	104.1/6.74	C_2,6_-H_2,6_ in etherified syringyl units (S)
G_2_	111.0/7.01	C_2_-H_2_ in guaiacyl units (G)
FA_2_	111.1/7.34	C_2_-H_2_ in ferulate (FA)
G_5_	114.4/6.73	C_5_-H_5_ in guaiacyl units (G)
PCA_3,5_	116.2/6.77	C_3_-H_3_ and C_5_-H_5_ in *p*-coumarate (PCA)
G_6_	119.0/6.82	C_6_-H_6_ in guaiacyl units (G)
PCA_2,6_	130.1/7.48	C_2,6_-H_2,6_ in *p*-hydroxyphenyl units (H)
PCA*_*a*_* and FA*_*a*_*	144.7/7.46	C*_*a*_*-H*_*a*_* in *p*-coumarate (PCA) and ferulate (FA)
**Associated carbohydrate and LCCs linkages**		
Ara_5_	61.9/3.52	C_5_-H_5_ in *a*-(1→4)-L-arabinofuranoside
X_5_	62.6/3.40	C_5_-H_5_ in β-(1→4)-D-xylopyranoside
X_*NR5*_	65.5/3.01,3.65	C_5_-H_5_ in β-(1→4)-D-xylopyranoside with non-ducing end
X_*NR4*_	69.5/3.24	C_4_-H_4_ in β-(1→4)-D-xylopyranoside with non-ducing end
X_2_	72.5/3.02	C_2_-H_2_ in β-(1→4)-D-xylopyranoside
X_3_	73.7/3.22	C_3_-H_3_ in β-(1→4)-D-xylopyranoside
X_4_	75.4/3.60	C_4_-H_4_ in β-(1→4)-D-xylopyranoside
Ara_3_	77.1/3.72	C_3_-H_3_ in *a*-(1→4)-L-arabinofuranoside
Ara_2_	81.6/3.89	C_3_-H_3_ in *a*-(1→4)-L-arabinofuranoside
*a*X_1_	92.0/4.88	C_1_-H_1_ in (1→4)-*a*-D-xylopyranoside
βX_1_	97.4/4.26	C_1_-H_1_ in (1→4)-β-D-xylopyranoside
X_1_	103.2/4.21	C_1_-H_1_ in β-(1→4)-D-xylopyranoside
Est+A′γ	65–62/4.5–4.0	γ-ester and A′γ in LCC
BE_1_	81.4/4.64	Benzyl ether (in primary OH of carbohydrate) in LCC
BE_2_	81.2/5.06	Benzyl ether (in secondary OH of carbohydrate) in LCC
PhGlc1	100.1/5.09	Phenyl glycoside-1 in LCC
PhGlc2	100.9/4.63	Phenyl glycoside-2 in LCC
PhGlc3	101.9/4.92	Phenyl glycoside-3 in LCC

In the side-chain regions (δ_C_/δ_H_ 90-50/6.0-2.5) of LCC and lignin spectra, both the substructures of β-O-4 (A) and resinols (β-5, C) can be easily determined on the basis of C*_α_*-H*_α_* signals at δ_C_/δ_H_ 71.8/4.87 and δ_C_/δ_H_ 86.8/5.49, respectively. In addition, the erythro forms of syringyl (S)-type β-O-4 substructures was identified at δ_C_/δ_H_ 86.1/4.11 (C*_β_*-H*_β_* correlations) in LCC-C-CR and LCC-L-CR spectra, indicating that substructure A could be linked to S units in the lignin unit of LCCs. Meanwhile, guaiacyl (G)-type β-O-4 substructures (C*_β_*-H*_β_* correlations at δ_C_/δ_H_ 83.9/4.30) was also identified in Lignin-CR spectra, indicating that both guaiacyl and syringyl units could be linked to β-O-4 in the lignin. The identification of β-O-4 and β-5 substructures is not beyond expectation since they are commonly shared by the LCCs/lignins from hardwood (Jiang et al., 2019). Our aforementioned results suggest that both LCC-C-CR and LCC-L-CR are composed of similar chemical substructures (Figure 1). To further delineate structural differences among the LCC and lignin preparations, the quantification of lignin substructures was determined (Table 4). We found that Lignin-CR showed highest amount of each substructure, with 34.1/100 Ar for β-O-4, 16.5/100 Ar for β-5 and 16.1/100 Ar for *a*,β-diaryl ether. The β-O-4 (10.2/100 Ar), β-5 (8.7/100 Ar), and *a*,β-diaryl ether (12.7/100 Ar) substructures in LCC-C-CR were less abundant than those in LCC-L-CR (15.9/100 Ar for β-O-4, 10.5/100 Ar for β-5 and 14.2/100 Ar for *a*,β-diaryl ether substructures). According to the published research findings, the amount of β-O-4 ranges from 21/100 Ar to 100/100 Ar in LCC preparations from bamboo (Dong et al., 2020), Ginkgo (Jiang et al., 2019), and Arundo donax Linn (You et al., 2015). The lower amount of β-O-4 substructure in LCC preparations from *C. retusus* tender leaf might be due to insufficient lignification, since the growth age is 1–3 months.

**TABLE 4:** The amount of lignin substructure and lignin-carbohydrate linkages in LCC-C-CR, LCC-L-CR, and Lignin-CR preparations (100 Ar).

Characteristics	LCC-C-CR	LCC-L-CR	Lignin-CR
**Lignin interunit linkages**			
β-O-4 aryl ethers (A)	10.2	15.9	34.1
phenylcoumarans, β-5 (C)	8.7	10.5	16.5
*a*,β-diaryl ether, *a*-O-4 (E)	12.7	14.2	16.1
S/G ratio^*a*^	0.63	0.44	0.39
**LCC linkages**			
benzyl ether (BE)	4.3	9.7	/
phenyl glycoside (PhGlc)	10.5	8.3	1.1
Est^b^	6.3	5.9	12.5
Total^c^	21.1	23.9	13.6

*^*a*^Calculated based on 2D HQSC spectra data using the formula: S/G = I_103–108_/6.4–7.0/(I_108–114_/_6.9–7.3_/2). ^*b*^Sum of LCCs γ-esters (Est) and γ-acylated β-O-4 aryl ether substructures. ^*c*^Sum of the amount of BE, PhGlc, and Est.*

In the aromatic regions (δ_C_/δ_H_ 160-90/8.0-6.0) of LCC-C-CR, LCC-L-CR and Lignin-CR spectra, the major correlation signals of syringyl unit (S) and guaiacyl unit (G) of lignin were identified. The signal appeared at δ_C_/δ_H_ 104.1/6.72 was attributed to the C_2,6_-H_2,6_ correlations of S unit. For the G unit, the correlations of C_2–_H_2_, C_5–_H_5_, and C_6–_H_6_ were observed by the signals at δ_C_/δ_H_ 111.0/7.01, 114.4/6.73, and 119.0/6.82, respectively. In LCC-C-CR, LCC-L-CR, and Lignin-CR, the respective S/G ratio was 0.63, 0.44, and 0.39, and these are lower than the S/G ratio of LCCs from Arundo donax Linn (0.65) and poplar (1.62) (Yuan et al., 2011; You et al., 2015). Taken together, the differences in the amount of substructures and S/G value between current and previous studies highlight that growth age may influence not only the contents of aryl ether linkages but also the S/G ratio of LCC/lignin in different biomass (Jiang et al., 2019).

The prominent signals attributed to *p*-coumarate (PCA) and ferulate (FA) structures were also observed in LCC-C-CR, LCC-L-CR and Lignin-CR, although they are typically undetectable in general woody biomass (Table 3). As pointed out by Jiang et al. (2019), both PCA and FA actively participate in the inchoate process of lignification. The FA provides the initiation sites for lignification in the cell wall, whereas monolignol conjugated PCA provides building bridge for lignin (Ralph, 2010). Therefore, the presence of the C-H correlations of PCA and FA emphasizes that *C. retusus* tender leaves used in this study are still in the process of lignification. This further supports our previous hypothesis regarding the impact of growth age on lignin chemical properties. It is noteworthy that the presence of PCA and FA structures also contribute to the antioxidant capability of LCC (Jiang et al., 2020a), suggesting the essential roles of PCA and FA in carrying out biological functions of LCC and lignin.

### Identification of Associated Carbohydrates and LCC Types in LCC Preparations

In the 2D HSQC spectra, multiple types of signals of associated carbohydrates in LCCs were also detected, including the β-D-xylopyranoside units (X), glucopyranoside units (Glu), and arabinofuranoside units (Ara) (Jiang et al., 2019; Dong et al., 2020). The cross-peaks for correlations of C_1_-H_1_, C_2_-H_2_, C_3_-H_3_, C_4_-H_4_, and C_5_-H_5_ in X can be found at δ_C_/δ_H_ 103.2/4.19, 72.5/3.03, 73.7/3.22, 75.6/3.63, and 62.6/3.40, respectively. In addition, the types of *a*-D-(1→4)-xylopyranoside units (*a*X) and β-D-(1→4)-xylopyranoside units (βX), which can be differentiated by the C_1_-H_1_ correlations at δ_C_/δ_H_ 92.0/4.88 and δ_C_/δ_H_ 97.0/4.26, were present in both LCC-C-CR and LCC-L-CR (You et al., 2015). Overall, the identification of associated carbohydrates in LCCs were in line with the results from our compositional analysis (Table 1).

The generally accepted lignin-carbohydrate linkages in LCC are benzyl ethers (BE), phenyl glycosides (PhGlc), and benzyl esters (Est) (Balakshin et al., 2007; Yuan et al., 2011). In Figure 1, the cross-peaks of PhGlc linkages were observed in the spectra of both LCC-C-CR and LCC-L-CR. In the region of δ_*C*_/δ_*H*_ 105-90/5.4-3.9, three kinds of PhGlc can be detected as indicated by the signals at δ_*C*_/δ_*H*_ 100.1/5.09 (PhGlc1), 100.9/4.63 (PhGlc2), and 101.5/4.79 (PhGlc3). However, only PhGlc3 was detected in Lignin-CR. Based on the quantification analysis (Table 4), LCC-C-CR showed a slightly higher amount of PhGlc (10.5/100 Ar) than LCC-L-CR (8.3/100 Ar) and Lignin-CR (1.1/100 Ar). This may be due to the higher total contents of glucan and xylan (52.0%) in LCC-C-CR, comparing to LCC-L-CR (29.8%). As reported by Balakshin et al. (2011) and Yuan et al. (2011), the predominant types of carbohydrates that are involved in the formation of phenyl glycoside linkages are glucan and xylan. For BE linkages, LCC spectra revealed the signals at δ_*C*_/δ_*H*_ 83.4/4.63 (BE1) and δ_*C*_/δ_*H*_ 83.2/5.14 (BE2), which are attributed to the C1-linkages between the a-position of lignin and primary OH groups of carbohydrates, and C2-linkages between the *a*-position of lignin and secondary OH groups of carbohydrates. As shown in Table 4, the amount of benzyl ether linkages in LCC-C-CR and LCC-L-CR were 4.3/100 Ar and 9.7/100 Ar, respectively. However, these signals were absent in Lignin-CR due to its high purity with only trace amount of carbohydrate. Generally, the benzyl ester linkage is observed by *a*-ester or γ-ester signal at δ_*C*_/δ_*H*_ 75/6.1 and δ_*C*_/δ_*H*_ 65-62/4.0-4.5 (Jiang et al., 2020b). In this study, no signals from *a*-ester structures could be detected in LCC and lignin preparations. This is similar to what was reported in Balakshin et al. (2011). Although γ-ester showed the signal at δ_*C*_/δ_*H*_ 64.5/4.19 in LCC-C-CR and LCC-L-CR, this could be attributed to the signal of γ-acylated lignin moiety (A′). As pointed out by Balakshin et al. (2011), the signal of LCC γ-esters can overlap with the signal from γ-acetyl of substructures A (A′). Those two overlapping signals could be distinguished using higher powered NMRs. The calculated γ-esters value in our LCC samples is the sum of benzyl ester in LCC and acetyl γ-esters in lignin. As demonstrated in Table 4, the amount γ-ester was more abundant in Lignin-CR (12.5/100 Ar), when compared to LCC-C-CR (6.3/100 Ar) and LCC-L-CR (5.9/100 Ar). Regardless the amount of γ-ester, we found that PhGlc was the predominant linkage in LCC-C-CR (49.8%), while BE was the predominant linkage in LCC-L-CR (40.1%). These findings are in accordance to previous report, in which You et al. (2015) demonstrated that carbohydrate-rich LCC and lignin-rich LCC derived from Arundo donax Linn were enriched in PhGlc (84.8%) and BE (96.6%) linkages, respectively.

### Antioxidant Activity Analysis of LCC and Lignin Preparations

Both lignin and carbohydrate have been implicated in scavenging free radicals *in vitro* and *in vivo* due to their antioxidant activities (Wang L. et al., 2019). We then examined if LCCs from *C. retusus* tender leaf exert similar antioxidant functions. In addition, the carbohydrate-free lignin preparation (Lignin-CR) was also included. The 2,2-diphenyl-1-picrylhydrazyl (DPPH) radicals and superoxide anion (O_2_^•–^) radicals were used as the reference substances to evaluate their scavenging capabilities *in vitro*.

**FIGURE 3:**
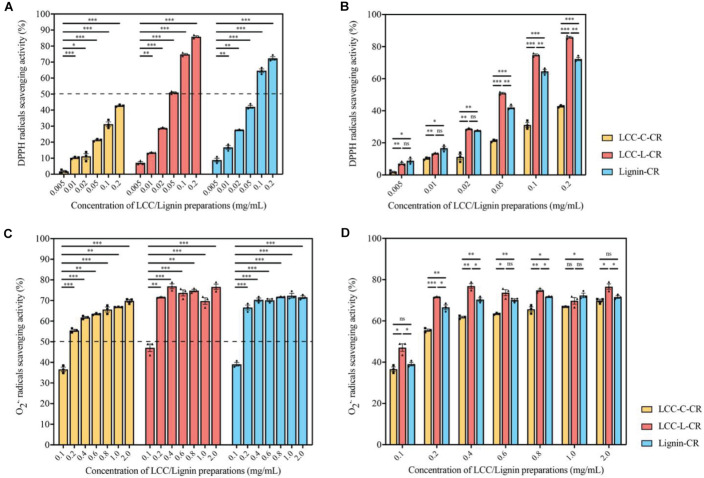
The LCC and lignin preparations showed antioxidant activities. **(A)** The DPPH radicals scavenging activities of LCC and lignin preparations were increased dose-dependently. *n* = 3 biological replicates. Each *n* represents an independent preparation of antioxidant assay samples. Error bars represent S.E.M. Statistical analysis was performed using two-tailed unpaired Student’s *t*-test. ^∗^ denotes *p* < 0.05, ^∗∗^ denotes *p* < 0.01, and ^∗∗∗^ denotes *p* < 0.001. **(B)** The LCC-L-CR elicited more potent scavenging capability than LCC-C-CR and Lignin-CR, especially when treated at higher concentrations. *n* = 3 biological replicates. Each *n* represents an independent preparation of antioxidant assay samples. Error bars represent S.E.M. Statistical analysis was performed using one-way ANOVA followed by *post hoc* Tukey’s test. ns denotes no significant difference, ^∗^ denotes *p* < 0.05, ^∗∗^ denotes *p* < 0.01, and ^∗∗∗^ denotes *p* < 0.001. **(C)** The scavenging capabilities of LCC and lignin preparations toward O_2_^•–^ were significantly enhanced when used at higher concentrations. *n* = 3 biological replicates. Each *n* represents an independent preparation of antioxidant assay samples. Error bars represent S.E.M. Statistical analysis was performed using two-tailed unpaired Student’s *t*-test. ^∗∗^ denotes *p* < 0.01 and ^∗∗∗^ denotes *p* < 0.001. **(D)** The O_2_^•–^ radical scavenging activity of LCC-L-CR was significantly higher than that of LCC-C-CR and Lignin-CR, except for treating at 0.6 and 1.0 mg/mL. *n* = 3 biological replicates. Each *n* represents an independent preparation of antioxidant assay samples. Error bars represent S.E.M. Statistical analysis was performed using one-way ANOVA followed by *post hoc* Tukey’s test. ns denotes no significant difference, ^∗^ denotes *p* < 0.05, ^∗∗^ denotes *p* < 0.01 and ^∗∗∗^ denotes *p* < 0.001.

As demonstrated in Figure 3A, the scavenging capabilities toward DPPH radicals were significantly enhanced when the treatment concentrations of LCC-C-CR, LCC-L-CR and Lignin-CR were dose-dependently increased. The scavenging activity of LCC-L-CR was significantly stronger than that of LCC-C-CR and Lignin-CR, especially when used at higher concentrations (Figure 3B). Meanwhile, LCC-L-CR showed an IC_50_ concentration (the concentration for quenching 50% of the initial radical) of 0.05 mg/ml, which was lower than that of LCC-C-CR (>0.2 mg/mL) and Lignin-CR (0.06 mg/ml) (Figure 3A). As for O_2_^•–^ radicals, the scavenging capabilities were also significantly upregulated upon treatment of higher concentrations of LCC-C-CR, LCC-L-CR, and Lignin-CR (Figure 3C). Compared to LCC-C-CR and Lignin-CR, the scavenging activity of LCC-L-CR was significantly stronger, except for treating at 0.6 and 1.0 mg/mL (Figure 3D). The IC_50_ value for O_2_^•–^ radicals scavenging activity in LCC-L-CR treatment group was 0.11 mg/mL, which was lower than the IC_50_ in LCC-C-CR (0.19 mg/mL) and Lignin-CR (0.14 mg/mL) treatment groups (Figure 3C). Taken together, these results indicate that the antioxidant activity of neat lignin could be further potentiated when a certain amount of carbohydrate was structurally incorporated. This is in agreement with a previous study reported by Zhang et al. (2018). In our obtained LCC preparations, both of them exhibit free radicals scavenging activities, while the scavenging capability of LCC-L-CR is higher than that of LCC-C-CR, especially toward DPPH radicals.

Together with chemical compositional data (Table 1), we found that LCC preparation with less amount of carbohydrates exhibited greater antioxidant potency than carbohydrate-rich LCC and neat lignin (Figure 3). However, in another study, Zhao et al. (2015) found that hemicellulose fractions with different monosaccharide composition and molecular weight distribution demonstrated superior antioxidant capacity than a lignin-rich fraction. The discrepancy in these findings might be attributed to the different amount of hydrogen bonds formed between polar groups in carbohydrates and free phenolic functionalities in lignin (Ugartondo et al., 2008). In the current study, the higher percentage of carbohydrate in LCC-C-CR could result in more hydrogen bonding and thus lead to decreased antioxidant potency.

**FIGURE 4:**
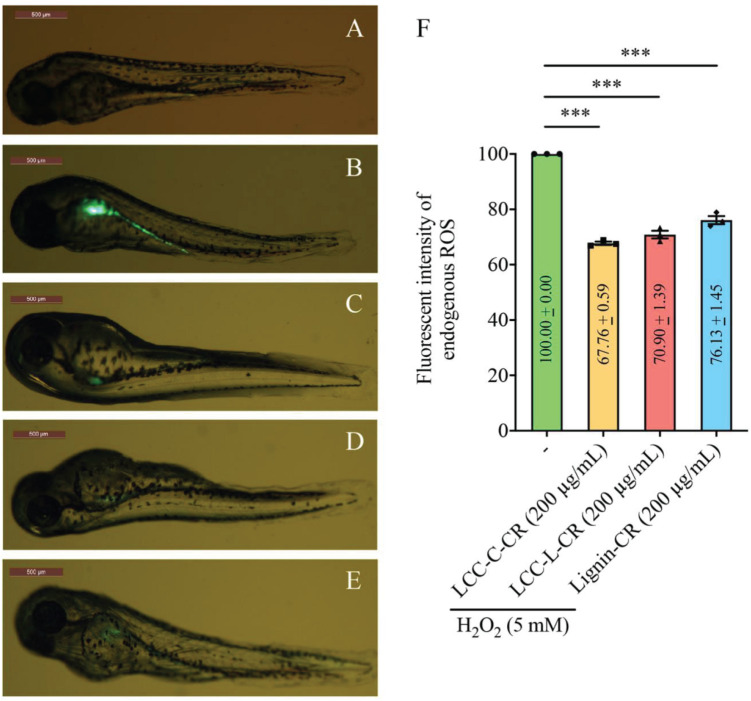
Both LCC and lignin preparations scavenged ROS production *in vivo*. The level of ROS production in zebrafish model was reflected by the intensity of green fluorescent signal in non-treatment **(A)**, H_2_O_2_-treated **(B)**, LCC-C-CR-treated **(C)**, LCC-L-CR-treated **(D),** and Lignin-CR-treated **(E)** groups. Scale bars: 500 μm. *n* = 3 biological replicates. Each *n* represents an independent preparation of fluorescence imaging samples. Only representative images were shown. **(F)** Quantification of the green fluorescent signal in panel **(B–E)**. At least four zebrafish were used in H_2_O_2_, LCCs or lignin-treated group from an independent experiment. Error bars represent S.E.M. Statistical analysis was performed using two-tailed unpaired Student’s *t*-test. ^∗∗∗^ denotes *p* < 0.001.

We next exploited a zebrafish model to evaluate the antioxidant activities of our LCC and lignin preparations *in vivo*. The non-treatment group (normal zebrafish) exhibited undetectable green fluorescent signal (acquired using confocal laser scanning microscopy) (Figure 4A). Once stimulated with H_2_O_2_, the endogenous ROS (green fluorescent signal) was generated and concentrated in the yolk sac and its extension in zebrafish body (Figure 4B). Intriguingly, when the fishes were treated with either LCC-C-CR (Figure 4C), LCC-L-CR (Figure 4D) or Lignin-CR (Figure 4E) prior to H_2_O_2_ stimulation, the fluorescent intensity was significantly suppressed (Figure 4F). It can therefore be concluded that both the isolated LCC and lignin preparations possess *in vivo* antioxidant activity to scavenge the production of endogenous ROS.

### Effect of LCC and Lignin Preparations on ATXN3Q71 Protein Aggregation

It has been previously reported that accumulation of oxidative species correlates with polyQ disease pathologies, and inhibition of oxidative stress attenuates the formation of polyQ protein aggregates and suppresses polyQ-induced cytotoxicities (Essa et al., 2019). Chang et al. (2014) demonstrated that geniposide, genipin and crocin from *Gardenia jasminoides* inhibit intracellular aggregation of polyQ protein via lowering the production of ROS in neuronal cells, suggesting an anti-protein aggregation activity of antioxidants from natural product. Given their antioxidant capabilities, we next tested if our LCC and lignin preparations would elicit any effect on modifying polyQ protein aggregation. A polyQ disease cell model was established with the overexpression of SCA type3 (SCA3) disease protein ATXN3Q71 (Ataxin-3 protein harboring 71 repeats of glutamine) in a human neuroblastoma cell line SK-N-MC (Chen Z. S. et al., 2018; Chen et al., 2019). The percentage of cells with polyQ protein aggregates was used as readout to evaluate the protein aggregation-modulatory effects of LCCs and lignin.

**FIGURE 5:**
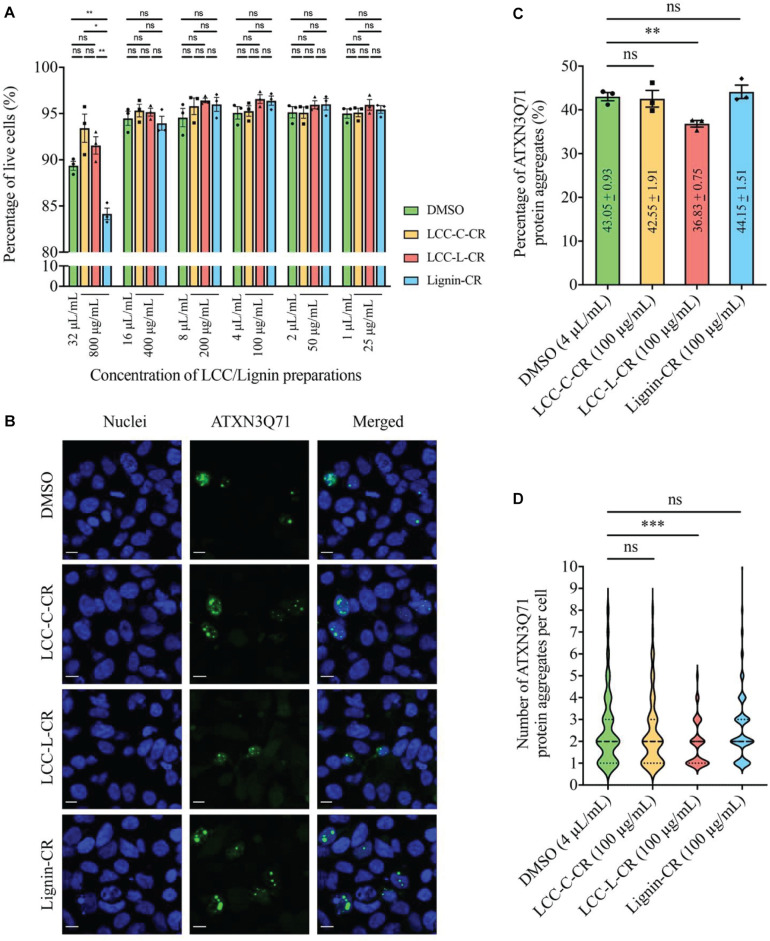
The lignin-rich LCC preparation showed anti-protein aggregation activity toward polyQ protein aggregates. **(A)** Cytotoxicities of LCC and lignin preparations were tested on human neuroblastoma SK-N-MC cells. No significant difference on the percentage of live cells was detected between DMSO, LCC-C-CR, LCC-L-CR, and Lignin-CR groups when the treatment ranges from 25 to 400 μg/mL. When treated at 800 μg/mL, the percentage of live cells in Lignin-CR group was significantly reduced. *n* = 3 biological replicates. Each *n* represents an independent preparation of cytotoxicity sample. Error bars represent S.E.M. Statistical analysis was performed using one-way ANOVA followed by *post hoc* Tukey’s test. ns denotes no significant difference, ^∗^ denotes *p* < 0.05 and ^∗∗^ denotes *p* < 0.01. **(B)** Treatment of LCC-L-CR, but not LCC-C-CR or Lignin-CR, suppressed the percentage of cells with ATXN3Q71 protein aggregates (green) in SK-N-MC cells. Cell nuclei (blue) were stained with DAPI. Scale bars: 50 μm. *n* = 3 biological replicates. Each *n* represents an independent preparation of fluorescence imaging samples. Only representative images were shown. **(C)** Quantification of the percentage of cells with ATXN3Q71 protein aggregates in panel **(B)**. At least 85 of ATXN3Q71-transfected cells were counted in DMSO, LCCs or lignin-treated group from an independent experiment. Error bars represent S.E.M. Statistical analysis was performed using two-tailed unpaired Student’s *t*-test. ns denotes no significant difference and ^∗∗^ denotes *p* < 0.01. **(D)** Quantification of the number of ATXN3Q71 protein aggregates per cell in panel **(B)**. At least 35 cells with the formation of ATXNQ71 protein aggregates were counted in DMSO, LCCs or lignin-treated group from an independent experiment. Dash lines indicate median, while dotted lines indicate 25th and 75th quartiles. Statistical analysis was performed using Mann–Whitney *U* test. ns denotes no significant difference and ^∗∗∗^ denotes *p* < 0.001.

The cytotoxicities of LCC and lignin preparations were first examined in SK-N-MC cells without expression of polyQ disease protein. As shown in Figure 5A, when treated at 800 μg/mL, the Lignin-CR group demonstrated significant lowered percentage of live cells compared to vehicle control (DMSO), LCC-C-CR and LCC-L-CR groups. Such reduction of cell viability was not observed when Lignin-CR was used ranging from 25 to 400 μg/mL, indicating the neat lignin fraction was slightly toxic to SK-N-MC cells, especially when treated at higher concentration. There is no significant difference on the percentage of live cells between DMSO-treated and LCC-treated groups, suggesting that obtained LCCs have low cytotoxicities and good biocompatibilities with SK-N-MC cells (Figure 5A).

Based on our cytotoxicity results, the two LCC preparations were further tested on the ATXN3Q71-expressing SK-N-MC cells, with the aim to evaluating their capabilities in modulating polyQ protein aggregation. The fluorescent images were captured by confocal laser scanning microscopy to visualize the formation of polyQ protein aggregates (Adegbuyiro et al., 2017). As demonstrated in Figure 5B, overexpression of expanded ATXN3Q71 protein led to the formation of intracellular protein aggregates (green fluorescent signal). We found that treatment with 100 μg/mL LCC-L-CR, but not LCC-C-CR or Lignin-CR, effectively suppressed the polyQ protein aggregation in ATXN3Q71-expressing cells (Figure 5B). The corresponding statistics (Figure 5C) showed that the percentage of cells with ATXN3Q71 protein aggregates was significantly downregulated from 43.1 to 36.8% upon treatment of LCC-L-CR, whereas no significant change was observed in the LCC-C-CR (42.6%) and Lignin-CR (44.2%) treatment groups. Meanwhile, the number of aggregated ATXN3Q71 protein (represented by green fluorescent dots) in each aggregates-containing cell was also determined. We found that treatment of LCC-L-CR, but not LCC-C-CR or Lignin-CR, significantly reduced the number of ATXN3Q71 protein aggregates per cell (Figure 5D). Moreover, treatment of LCC-C-CR, LCC-L-CR or Lignin-CR did not significantly alter the percentage of ATXN3Q71-transfected cells, suggesting that the suppression of ATXN3Q71 protein aggregates mediated by LCC-L-CR was not due to the perturbation of the transfection efficiency of *pAcGFP-ATXN3Q71* plasmid in SK-N-MC cells (Supplementary Figure 2).

These results therefore suggest an anti-protein aggregation effect of LCC-L-CR toward ATXN3Q71 protein aggregates. The difference in the capabilities in suppressing ATXN3Q71 protein aggregation among LCC-L-CR, LCC-C-CR, and Lignin-CR might be due to the higher antioxidant activity of LCC-L-CR (Figure 3). Overall, the lignin-rich LCC, but not carbohydrate-rich LCC and neat lignin, isolated from *C. retusus* tender leaves showed sufficient antioxidant and anti-protein aggregation activities.

## Conclusion

In the current study, we managed to isolate lignin-rich LCC (LCC-L-CR), carbohydrate-rich LCC (LCC-C-CR), and lignin (Lignin-CR) from the hot-water extracted residual solids of *C. retusus* tender leaves. Both LCC preparations contain all types of LCC linkages but differ in abundance. The LCC-L-CR showed superior antioxidant potency, which is believed to be the driving force behind its pronounced capability to suppress the intracellular aggregation of polyQ disease protein. In conclusion, this is the first report to highlight the anti-protein aggregation activity of a lignin-rich LCC isolated from *C. retusus* tender leaves, and this provides a new angle to investigate the LCC’s biological functions. More importantly, our findings will contribute to the future studies of similar biological macromolecules in the biological and bioengineering fields.

## Data Availability Statement

All datasets presented in this study are included in the article/Supplementary Material.

## Ethics Statement

The animal study was reviewed and approved by the Animal Ethics Committee of Nanjing Drum Tower Hospital, The Affiliated Hospital of Nanjing University Medical School (Permit number: 2019AE01018).

## Author Contributions

WP and ZC: investigation. HC and CL: supervision. CH and LZ: writing – original draft. ZC and CH: writing – review and editing. All authors contributed to the article and approved the submitted version.

## Conflict of Interest

The authors declare that the research was conducted in the absence of any commercial or financial relationships that could be construed as a potential conflict of interest.
