# Supplementary material for: Isolation and Identification of a Novel Anti-protein Aggregation Activity of Lignin-Carbohydrate Complex From Chionanthus retusus Leaves
Source: Front Bioeng Biotechnol. 2020 Sep 25;8:573991. doi: 10.3389/fbioe.2020.573991 (PMC7546364; doi:10.3389/fbioe.2020.573991)
Supplement: Supplementary file 1 [file Data_Sheet_1.pdf]

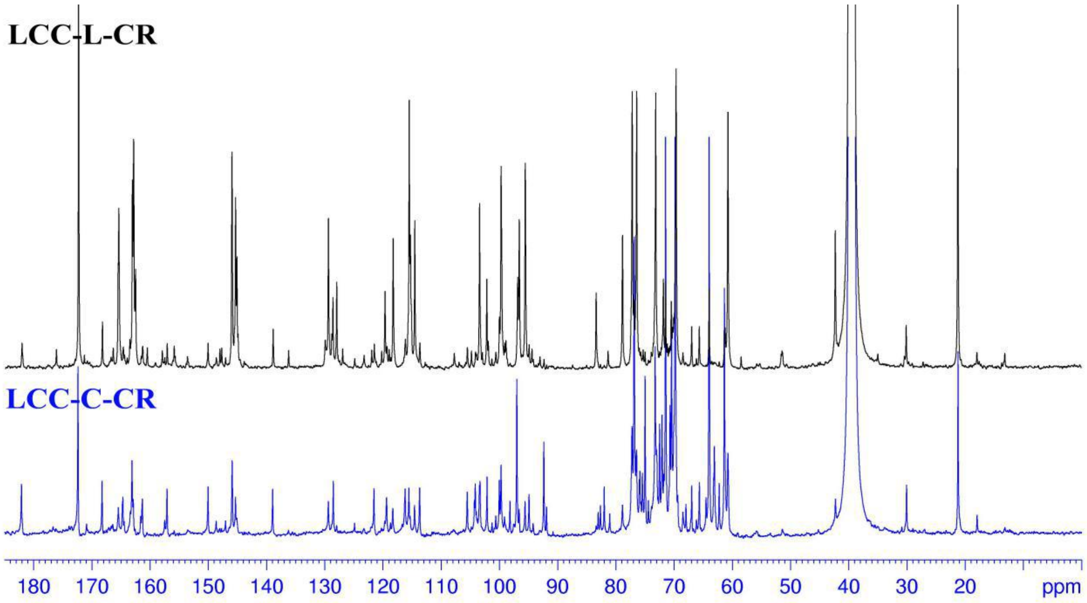

Supplementary Figure 1 ^{13}C NMR spectra of LCC-L-CR and LCC-C-CR (using DMSO-d6 as the dissolving reagent)

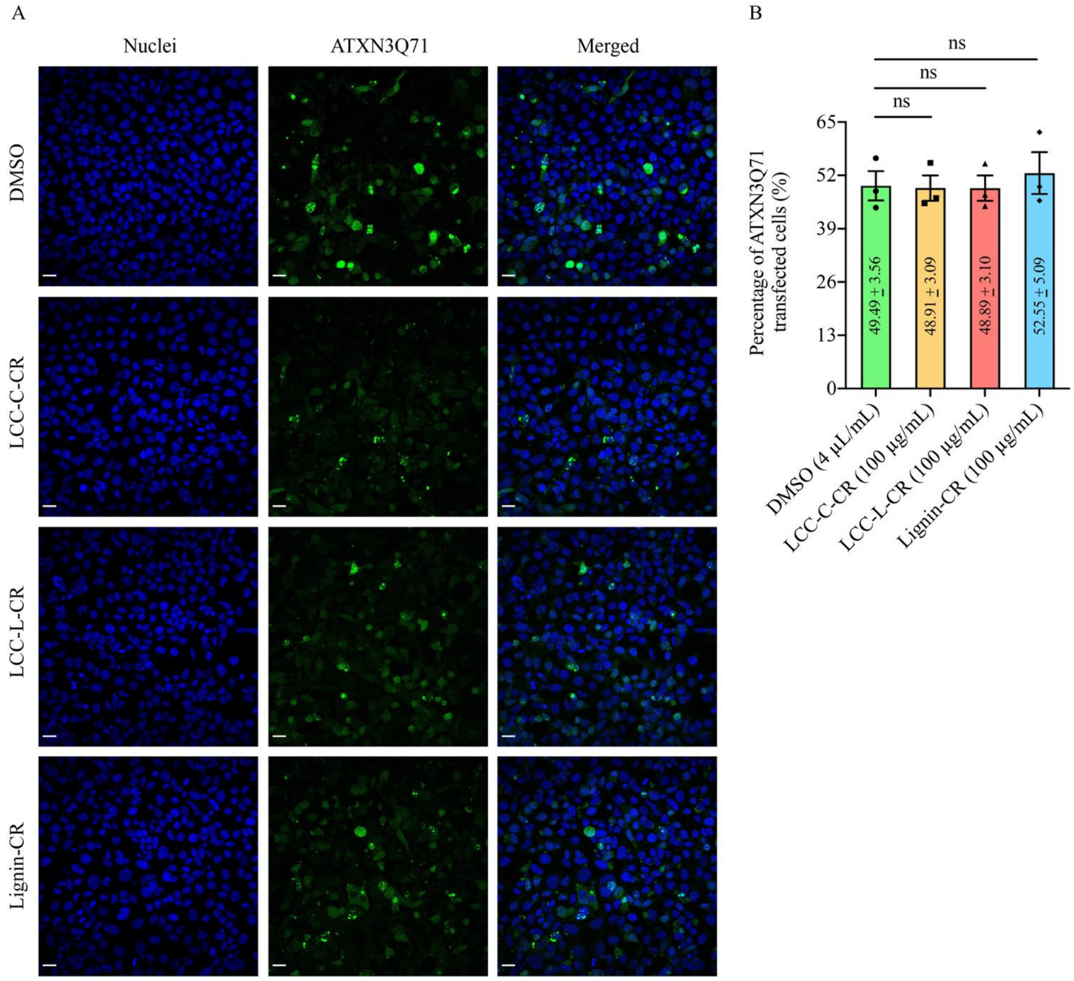

Supplementary Figure 2. Treatment of LCC or Lignin-CR preparations did not affect the transfection efficiency of *pAcGFP-ATXN3Q71* plasmid. **(A)** The transfection efficiency of *pAcGFP-ATXN3Q71* plasmid, as demonstrated by the percentage of cells positive for ATXN3Q71 protein expression (green) in SK-N-MC cells, was not altered upon treatment of LCC or Lignin-CR preparations. Cell nuclei (blue) were stained with DAPI. Scale bars: 100 μm. n = 3 biological replicates. Each n represents an independent preparation of imaging sample. Only representative images were shown. **(B)** Quantification of the percentage of ATXN3Q71 transfected cells in panel **(A)**. At least 1400 cells were counted in DMSO, LCCs or Lignin-CR-treated group from an independent experiment. Error bars represent S.E.M.. Statistical analysis was performed using two-tailed unpaired Student's t-test. ns denotes no significant difference.
